# An objective tropical Atlantic sea surface temperature gradient index for studies of south Amazon dry-season climate variability and change

**DOI:** 10.1098/rstb.2007.0024

**Published:** 2008-02-11

**Authors:** Peter Good, Jason A Lowe, Mat Collins, Wilfran Moufouma-Okia

**Affiliations:** Met Office Hadley CentreFitzRoy Road, Exeter, Devon EX1 3PB, UK

**Keywords:** Amazon die-back, tropical forest, tropical Atlantic, climate change impacts, climate model uncertainty, climate projections

## Abstract

Future changes in meridional sea surface temperature (SST) gradients in the tropical Atlantic could influence Amazon dry-season precipitation by shifting the patterns of moisture convergence and vertical motion. Unlike for the El Niño-Southern Oscillation, there are no standard indices for quantifying this gradient. Here we describe a method for identifying the SST gradient that is most closely associated with June–August precipitation over the south Amazon. We use an ensemble of atmospheric general circulation model (AGCM) integrations forced by observed SST from 1949 to 2005. A large number of tropical Atlantic SST gradient indices are generated randomly and temporal correlations are examined between these indices and June–August precipitation averaged over the Amazon Basin south of the equator. The indices correlating most strongly with June–August southern Amazon precipitation form a cluster of near-meridional orientation centred near the equator. The location of the southern component of the gradient is particularly well defined in a region off the Brazilian tropical coast, consistent with known physical mechanisms. The chosen index appears to capture much of the Atlantic SST influence on simulated southern Amazon dry-season precipitation, and is significantly correlated with observed southern Amazon precipitation.

We examine the index in 36 different coupled atmosphere–ocean model projections of climate change under a simple compound 1% increase in CO_2_. Within the large spread of responses, we find a relationship between the projected trend in the index and the Amazon dry-season precipitation trends. Furthermore, the magnitude of the trend relationship is consistent with the inter-annual variability relationship found in the AGCM simulations. This suggests that the index would be of use in quantifying uncertainties in climate change in the region.

## 1. Introduction

The characteristics of dry-season precipitation are important, in combination with land-use changes, in determining the fate of tropical vegetation and peatlands (e.g. Aragão *et al*. 2007; Li *et al*. 2007; Salazar *et al*. 2007). In the Amazon region, precipitation seasonality is dominated by north–south migrations of the intense rain band fuelled by moisture-laden trade winds (e.g. Xie & Carton 2004). One way in which sea surface temperature (SST) can influence this is through temporal fluctuations in the meridional tropical Atlantic SST gradient. Such changes shift the marine Intertropical Convergence Zone (ITCZ) to the warmer hemisphere, with precipitation impacts extending over tropical South America (e.g. Nobre & Shukla 1996). In boreal summer (dry season for much of the Amazon), model studies (Knight *et al*. 2006; Harris *et al*. 2008) have shown that relative warming (cooling) in the north (south) tropical Atlantic can cause drying over the Amazon south of the equator. It has been suggested that an anomalously warm tropical north Atlantic contributed to the 2005 Amazon drought (Marengo 2006). The mechanisms of this dry-season influence have not been explored in detail, but the tropical Atlantic is the main external source of moisture for the Amazon north of 10° S in all seasons (Rao *et al*. 1996). A meridional ITCZ shift corresponds to a shift in the trades, and hence in the latitude of peak moisture transport. This is seen in a seasonal decline from July to October in westward moisture transport (Rao *et al*. 1996) to the Amazon near 8° S (continental rainfall increases in the same region from July to October due to the changing solar insolation pattern; Xie & Carton 2004). Fluctuations in the meridional SST gradient are a dominant form of tropical Atlantic variability (TAV) over inter-annual to multidecadal time scales (Xie & Carton 2004). Therefore, future changes in the tropical Atlantic meridional gradient are one possible driver of change for the Amazon rainforest and peatlands, with impacts up to global scale through carbon cycle feedbacks (Cox *et al*. 2000, 2004; Li *et al*. 2007).

Intercomparisons between model projections and validations against observations are important components of the process needed to formally quantify risks of different outcomes in the region. This requires quantification of the meridional SST gradient, but it is not clear how this should be done. There are no standard objective indices of tropical Atlantic meridional gradient, although many have been proposed (e.g. Servain *et al*. 1999; Chiang *et al*. 2002; Czaja *et al*. 2002). This is partly because inter-annual variability in this region is associated with more than one SST mode (e.g. Xie & Carton 2004). Also, the mechanism of variability considered here involves forcing of the atmosphere by anomalous SST gradients, rather than anomalous SST (see Xie & Carton 2004). Therefore, maps of correlations between Amazon precipitation and gridpoint Atlantic SST anomalies might be hard to interpret in terms of the SST gradients driving those precipitation anomalies. Differences in spatial patterns of inter-annual SST variability simulated by different coupled ocean–atmosphere climate models are a further problem. Therefore, indices based on the first few modes of TAV may be hard to interpret in a consistent manner when we are interested in a specific regional rainfall impact.

Here, we systematically search for the tropical Atlantic SST gradients most strongly associated with south Amazon dry-season precipitation using results from an atmosphere-only model forced by observed SST.

## 2. Model data and methods

We use the results from atmospheric general circulation model (AGCM) simulations forced by the HadISST1 reconstruction of observed global sea surface temperature and sea ice concentration (Rayner *et al*. 2003), from 1949 to 2005. We integrated the Hadley Centre HadAM3 (Pope *et al*. 2000), that is the atmospheric component of the coupled atmosphere–ocean general circulation model HadCM3 (Gordon *et al*. 2001). It has 19 hybrid vertical levels with a horizontal resolution of 2.5° latitude by 3.75° longitude. An ensemble of four simulations was performed, each run identical except for the atmospheric and land surface initialization (taken from different parts of a HadCM3 control experiment). Analysis of variance indicates that forced variability accounts for 80% of the ensemble mean south Amazon precipitation variance for this season (50% in each ensemble member). We quote ensemble mean results for June–August (JJA). The Amazon Basin mask was taken from the total runoff integrating pathways (TRIP; Oki & Sud 1998) river routing model, available online from http://hydro.iis.u-tokyo.ac.jp/∼taikan/TRIPDATA/TRIPDATA.html. South Amazon Basin means were estimated by interpolating model data to the 1° TRIP grid, before averaging over the basin south of the equator.

A basic validation was performed using gridded surface precipitation observations from the University of Delaware (Willmott & Matsuura 1995; UDel_AirT_Precip data provided by the NOAA/OAR/ESRL PSD, Boulder, CO, USA, from their website at http://www.cdc.noaa.gov/) for 1950–1999, converted to south Amazon JJA averages as for the model. The model reproduces these observations to within uncertainty from stochastic atmospheric variability (correlation coefficient between the model ensemble mean and the observations is 0.56). The coupled version (HadCM3) has a relatively good simulation of the Amazon dry-season length (Li *et al*. 2006).

To demonstrate how our results apply to climate change, projections from 36 different coupled (ocean–atmosphere) climate models were also used. These were forced by a scenario of CO_2_ increasing by 1% yr^−1^ compounded until the year 1970 (by which time the concentration has doubled) and is then held constant. Seventeen of the models are variants of HadCM3 in which key atmospheric parameters are perturbed. These are as described by Collins *et al*. (2006) except for: (i) a different technique was used to determine the flux adjustment terms, giving significantly reduced high-latitude SST biases and (ii) different combinations of parameters were chosen to explore a wider range of surface and atmospheric feedbacks under climate change. The remaining 19 models (table 1) are from 15 different international institutions, the results made available as part of the Climate Model Intercomparison Project (CMIP3). To quantify trends, we use the rate of change, measured by a least-squares linear fit to the first 80 years of model data.

A previous study by Rowell (2001) used a systematic search for spatial SST gradients associated with Sahel precipitation. Rowell (2001) defined zonal and meridional SST gradients at each ocean gridpoint as the difference between SST averaged over two boxes. The size and spacing of the two boxes were fixed. Here we take a further step by also allowing the spacing between the boxes and the orientation of the gradient to vary. We did this by randomly choosing a large number (15 000) of pairs of boxes within the tropical Atlantic. Boxes containing more than 40% land were rejected. For the present study, the box sizes were fixed, at 25° longitude by 15° latitude. For each pair of boxes, a JJA SST gradient index was calculated as the difference between the respective area means. The correlation coefficient was calculated between that index and JJA southern Amazon precipitation. The results are summarized by examining the locations of those indices that correlate most strongly with JJA southern Amazon precipitation.

## 3. Results

Figure 1 introduces the characteristics associated with south Amazon precipitation anomalies in JJA. The cause of the dry season is illustrated by the location of the intense continental rainband north of the equator in JJA. This is emphasized in figure 1*b*, which shows the large meridional gradient in climate mean precipitation during this season. This large gradient means that regional precipitation will be sensitive to meridional shifts in the associated rainband. Underlying this seasonal mean picture is a steady sub-seasonal migration of the location of this rainband (Xie & Carton 2004). The colours in figure 1*a* provide some basic evidence for mechanisms of variability. JJA south Amazon precipitation anomalies are associated with a regional north–south dipole in precipitation (with a slight tilt), indicating a general shift in convection to the north–northeast, but also an intensification of the marine ITCZ in the east. Broadly, a north–south dipole is consistent with the concept of a meridional shift driven by SST gradients. The strongest correlations roughly follow the 3 mm d^−1^ climatological precipitation contour. The anomalies in south basin-averaged precipitation are dominated by anomalies between the equator and 10° S (the largest correlations in figure 1*a*), corresponding to the steep gradient in climatological precipitation (figure 1*b*). A broadly meridional gradient is indeed seen in the correlations between JJA south Amazon precipitation and SST anomalies (colours in figure 2). It is notable that the southern patch of associated (negative) SST correlations off tropical Brazil is much more localized than the positive correlations to the north.

Figure 2 also summarizes the main results of the SST gradient search (described in §2), showing the locations of the 100 indices with the strongest correlation with JJA south Amazon precipitation in the atmosphere model simulations. These identify an approximate meridional gradient centred near the equator. This is consistent with current physical understanding, being both a preferred form of tropical Atlantic SST variability and the one able to modify southern Amazon dry-season precipitation. The southern boxes are tightly clustered in a small area, while the northern boxes spread over a larger area. This is consistent with the aforementioned small area of anomaly correlations in the south. We conclude that it is relatively easy to define the southern box of our index. Our definition (20–40° W, 5–25° S) is marked in figure 2*a*. Figure 2*b* provides a check of the robustness of this selection, by plotting the southern box centres of the 2000 indices most strongly correlated with south Amazon precipitation. The points cluster around a well-defined region where the local SST is highly correlated with SST averaged over our chosen south index box. The box location, near to both the equator and the west coast, means that it is very likely the part of this SST mode most strongly influencing Amazon precipitation.

Having chosen the southern box of our index, the next step is to pick the northern box. At each gridpoint and for each year, the SST anomaly is calculated with respect to SST averaged over the southern box. Figure 3 shows the correlation coefficients between these anomalies and JJA south Amazon precipitation. If this correlation is large at a given gridpoint, this means that the difference between local SST and SST averaged over the southern box is strongly associated with JJA south Amazon precipitation. In other words, that gridpoint is a candidate for part of the north box of our index, given the prior choice of the southern box. (The weak positive correlations found almost everywhere appear by construction.) The strongest correlations form a near-zonal band in the north tropical Atlantic between 5 and 25° N. The strongest correlations are near the upwelling region off West Africa (roughly corresponding to the longitude of maximum SST variability), consistent with the gradient search results in figure 2. However, the westward decrease in correlation coefficient is not significant, and a larger region for area averaging might be more robust when applied to other contexts (e.g. to coupled climate models). Thus, we select a box extending across most of the basin (15–70° W, 5–25° N; figure 3). The northern box centres of the 2000 indices most strongly correlated with south Amazon precipitation all fall within or near to our chosen northern box (not shown), again suggesting that it is a robust choice. Notably, the region of the highest correlations does not quite correspond with those in figure 2, being more zonal, and further south near the east coast. This illustrates the point that patterns of SST anomalies may be hard to interpret in terms of anomalous SST gradients. The chosen index is the difference (north–south box averages), henceforth labelled TAGjja (Tropical Atlantic SST Gradient for JJA).

Figure 4 tests the chosen index, showing its correlation coefficient with gridded precipitation. This is quite similar to the pattern in figure 1, suggesting that the index is capturing much of the SST-forced variability associated with south Amazon dry-season precipitation. TAGjja is also significantly correlated with observed south Amazon dry-season precipitation (*r*=−0.60, compared with *r*=−0.71 between the TAGjja and the AGCM ensemble mean precipitation). This further strengthens the case for an Atlantic meridional gradient influence on the south Amazon dry season.

## 4. Discussion

The strong influence of meridional SST gradient variability on JJA precipitation shown in the results is perhaps surprising, given that the first empirical orthogonal function of tropical Atlantic precipitation variability during this season is associated with a different mode of variability, the Atlantic Niño (Kushnir *et al*. 2006), and that the ITCZ shows weaker meridional shifts in JJA than in austral summer/autumn (Chiang *et al*. 2002; Kushnir *et al*. 2006). However, the large meridional gradient in climate mean precipitation across the Amazon Basin (figure 1*b*) suggests a strong sensitivity to meridional shifts in the rainband. This may occur via shifts in moisture supply from the Atlantic, driven by SST gradient anomalies, although further studies of mechanisms in this season are required. The Atlantic Niño is likely to play some role (perhaps evidenced in the small patch of positive correlation in the Gulf of Guinea in figure 1*a*), although its direct influence is probably mostly limited to the north Amazon (Kushnir *et al*. 2006).

The SST gradients identified as having maximum correlation with Amazon precipitation (figure 2) correspond, perhaps unsurprisingly, with the only part of the Atlantic where a perfectly meridionally orientated cross-equatorial gradient is possible (due to the configuration of the coastline). In addition to this, the observation that the southern box of our meridional SST gradient was more localized and easier to define than the northern box may be due to a combination of factors. In JJA, SST variability in the central and eastern parts of the southern deep tropical Atlantic is dominated by the Atlantic Niño, which is more strongly associated with precipitation anomalies over the north Amazon (Kushnir *et al*. 2006). Our study region is on the southern flank of the continental ITCZ, which may perhaps mean that SST to the south has a strong controlling influence.

The chosen index may be regarded as close to a best estimate for this atmospheric model and is consistent with known physical mechanisms. The use of AGCM results to define this index offers a better ratio of signal to noise than would be possible using observed precipitation. The use of multiple AGCM integrations with different initial conditions allows for the quantification and minimization of the influence of stochastic atmospheric variability. These results are aimed primarily at climate model studies. On the other hand, forcing by observed SST means that the results should provide a relatively stable reference point, since the observed SST record will not change (except through improvements in reconstruction methodologies) and allows observational validation. It would be valuable to extend the analysis to results from different atmospheric models to assess robustness to model formulation. In principle, a similar analysis could have been applied to the climate model projections themselves, but since climate model projections change as models are improved, such results would not form a stable reference point from which to define a robust index.

By working with SST gradients rather than SST anomalies, this analysis has focused more closely on the action of a specific known mechanism of SST influence on precipitation. The nature of the random gradient search means that the results are harder to visualize. Potentially, such results could be further summarized using clustering or singular value decomposition techniques.

## 5. Applying the index to coupled model climate projections

We made an initial test of the importance of our derived index (TAGjja) in the context of anthropogenic climate change. First, linear regression was used to establish a relationship between TAGjja and JJA south Amazon precipitation, using the AGCM results only. Then, the projected 80-year trends in TAGjja and JJA south Amazon precipitation, under the scenario of increasing CO_2_, were calculated for each of the 36 different coupled climate models. Both the inter-annual AGCM regression and the multi-model trend projections are shown in figure 5. Although the scatter is reasonably large (expected, given that the models have large differences in tropical ITCZ location), it is clear that the crosses scatter around the linear regression from the AGCM results. Indeed, the solid line is very close to the optimal linear fit to the coupled model results. We emphasize that the solid line and the crosses are entirely independent: the former being based on inter-annual variability in an AGCM forced by observed SST, and the latter representing multidecadal climate change results from different coupled ocean–atmosphere models. This suggests that uncertainty in projected changes in the meridional Atlantic SST gradient is an important form of coupled model uncertainty of future changes in dry-season south Amazon precipitation. Second, TAGjja seems to be a good way of quantifying this gradient, in the context of both variability and climate change, and would be of use in formally quantifying uncertainty in predictions.

The impact of this mechanism is expected to be the largest between 10° S and the equator (figure 4). This overlaps the peatland region of Amazonia (red dashed rectangle, figure 4). Li *et al*. (2007) identified this region as a potentially contributing to important carbon climate feedbacks, but with substantial uncertainty due to model differences in projected dry-season precipitation change.

Salazar *et al*. (2007) used a potential vegetation model forced by climate change projections from 15 of the models participating in CMIP3. They found substantial model differences in potential vegetation changes over the Amazon between 10° S and the equator, ranging from minimal change to near-complete conversion to savannah.

Both the importance of meridional SST gradient change in terms of impacts on vegetation, and the mechanisms behind the range of model projections in this gradient need to be investigated further in order to better understand climate change impacts on the Amazon.

## Figures and Tables

**Figure 1:**
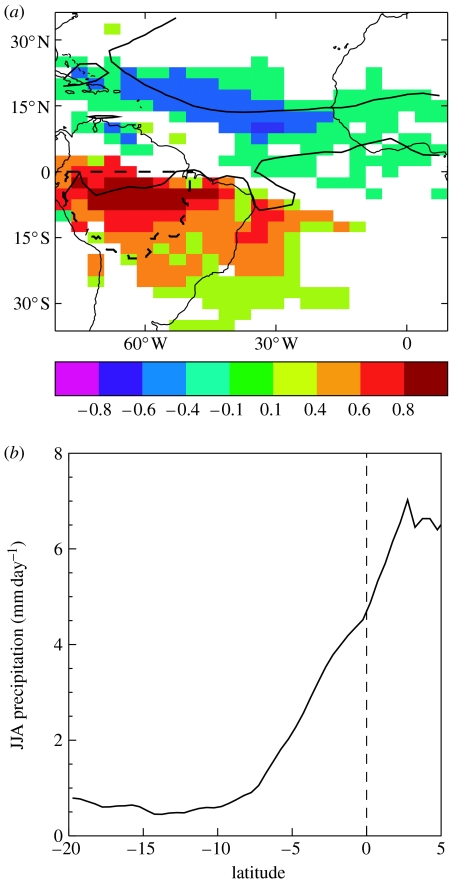
(*a*) Correlation coefficient for JJA between southern Amazon precipitation and gridpoint precipitation (masked where not significant at the 5% level according to the Mann–Kendall rank correlation test). Climatological ITCZ marked by 3 mm d^−1^ contour. Dashed line marks southern Amazon region. (*b*) Climate mean JJA precipitation zonally averaged across the Amazon Basin. The dashed line highlights the equator.

**Figure 2:**
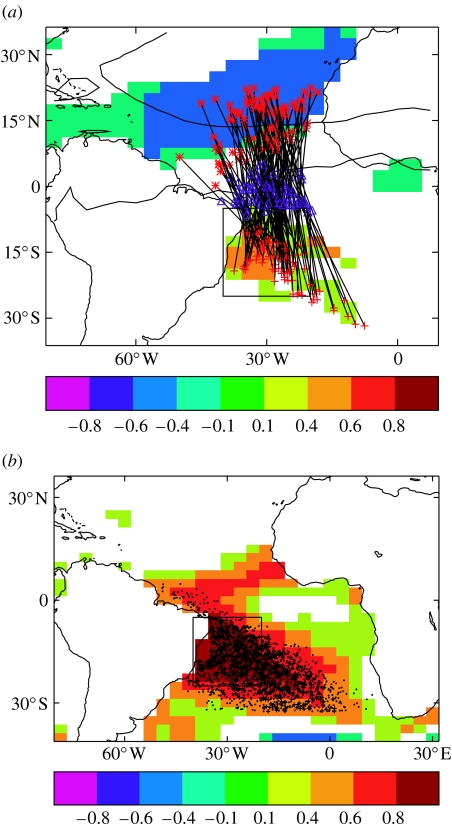
(*a*) Correlation coefficient for JJA between southern Amazon precipitation and gridpoint SST. The symbols connected by straight lines summarize the locations of the 100 indices with the strongest correlation with southern Amazon precipitation. For each index, a red asterisk (cross) marks the centre of the north (south) box, and a blue triangle marks the midpoint between two boxes. The black rectangle marks the chosen southern box. (*b*) Correlation coefficient for JJA between SST averaged over the rectangle shown, and gridpoint SST. Black points indicate the southern box centres of the 2000 indices with the strongest correlation with southern Amazon precipitation.

**Figure 3:**
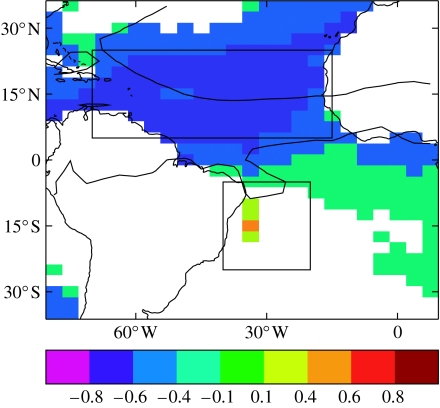
Choosing the northern box. Correlation coefficient for JJA between the gridpoint SST anomaly with respect to SST averaged over southern box, and southern Amazon precipitation. The northern and southern boxes of the chosen index are marked.

**Figure 4:**
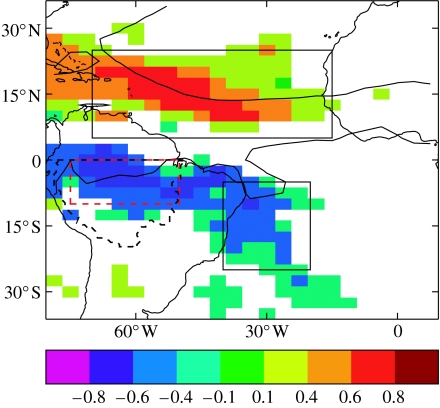
Correlation coefficient between TAGjja and gridpoint precipitation. Red dashed rectangle indicates the peatland region of Amazonia (Li *et al*. 2007).

**Figure 5:**
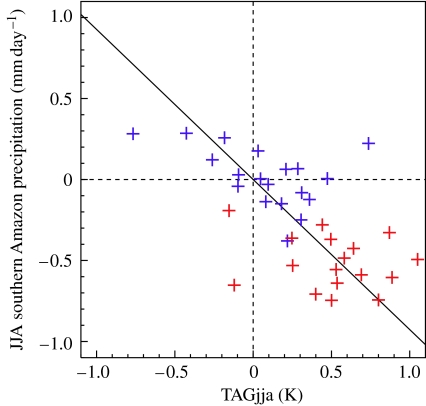
Solid line: linear regression between JJA southern Amazon precipitation and TAGjja, based on the AGCM results. Symbols: 80-year trend projections in the 1% experiment from 36 different coupled models. Each symbol represents one model (blue, AR4; red, perturbed versions of HadCM3). The solid line and the symbols are entirely independent.

**Table 1:** List of climate models from CMIP3. (Model documentation available via the PCMDI website (http://www-pcmdi.llnl.gov/).)

modelling group	IPCC I.D.
Bjerknes Centre for Climate Research, Norway	BCCR-BCM2.0
Météo-France/CNRM, France	CNRM-CM3
CSIRO Atmospheric Research, Australia	CSIRO-Mk3.0
GFDL, NOAA, USA	GFDL-CM2.0
	GFDL-CM2.1
GISS, NASA, USA	GISS-EH
	GISS-E-R
LASG/Institute of Atmospheric Physics, China	FGOALS-g1.0
Institute for Numerical Mathematics, Russia	INM-CM3.0
Institut Pierre Simon Laplace, France	IPSL-CM4
CCSR/NIES/FRCGC, Japan	MIROC3.2(hires)
	MIROC3.2(medres)
MPI, Meteorology, Germany	ECHAM5/MPI-OM
Meteorological Institute of the University of Bonn (Germany), Institute of KMA (Korea), and Model and Data Group	ECHO-G
Meteorological Research Institute, Japan	MRI-CGCM2.3.2
NCAR, USA	CCSM3
NCAR, USA	PCM1
Hadley Centre for Climate Prediction and Research, UK Met Office	UKMO-HadCM3
	UKMO-HadGEM1
